# Venereal Diseases

**Published:** 1891-05

**Authors:** L. P. Foster

**Affiliations:** Minneapolis, Minn.


### VENEREAL DISEASES.

L. P. Foster, M. D., Minneapolis, Minn.

To what extent do the old school cure their cases of venereal diseases? Five years ago a young woman contracted syphilis, and was treated by the regular school and pronounced cured. Three weeks ago a young man cohabited with her, and within ten days thereafter presented himself to me with a well-developed case of syphilitic ulcer and a venereal discharge. He accused the young woman of being impure; she denied the charge, and submitted to an examination of an old school expert and pronounced free from any venereal taint, and was given a certificate of this fact.

Seven years ago a young woman contracted gonorrhœa from her husband, and was treated by the old school and cured.

A few weeks ago a young man cohabited with her, she being now a widow, and within ten days presented himself to me with a well-developed case of gonorrhœa, and yet the old school pronounced this woman cured. If this is a cure, what kind is it?

